# Konzeptualisierung und Messung der Qualität der adaptiven Lernunterstützung in Lernsituationen mit mathematischen Regelspielen im Kindergarten. Eine Studie in Deutschland und der Schweiz

**DOI:** 10.1007/s13138-021-00195-2

**Published:** 2022-01-01

**Authors:** Anuschka Meier-Wyder, Andrea Wullschleger, Anke Lindmeier, Aiso Heinze, Miriam Leuchter, Franziska Vogt, Elisabeth Moser Opitz

**Affiliations:** 1 Interkantonale Hochschule für Heilpädagogik, Zürich, Schweiz; 2 Institut für Erziehungswissenschaft, Universität Zürich, Zürich, Schweiz; 3 Universität Koblenz-Landau, Landau, Deutschland; 4 Friedrich-Schiller Universität Jena, Jena, Deutschland; 5 Leibniz-Institut für die Pädagogik der Naturwissenschaften und Mathematik, Kiel, Deutschland; 6 Institut für Lehr- und Lernforschung, Pädagogische Hochschule St. Gallen, St. Gallen, Schweiz

**Keywords:** Unterstützungsqualität, Adaptive mathematische Lernunterstützung, Messinstrument, Kindergarten, Videorating, Quality of learning support, Adaptive mathematical learning support, Measuring instrument, Pre-school, Video-rating, B11, C51, C71, D41, M11, Q61

## Abstract

Die Qualität der adaptiven Lernunterstützung ist wichtig für die Förderung des mathematischen Lernens von Kindergartenkindern. Entsprechend bedarf es geeigneter Instrumente zur Bewertung der Planung, Durchführung und Reflexion von mathematischen Lernangeboten im Kindergarten. Bestehende Instrumente berücksichtigen vor allem die mikro-adaptive (mathematische) Lernunterstützung, die den Fokus auf die Interaktion zwischen der Fachkraft und den Kindern legt. Die Qualität der makro-adaptiven (mathematischen) Lernunterstützung (Planung und Reflexion von Lernsituationen) wurde im Kindergarten bis jetzt noch nicht umfassend untersucht. Sie gewinnt jedoch im Hinblick auf die zunehmende Bedeutung von Bildungsplänen im Kindergarten und der damit verbundenen Förderung von fachbezogenen Kompetenzen an Bedeutung.

Im Artikel wird erstens die Qualität von (mathematischer) Lernunterstützung in Regelspielsituationen im Kindergarten konzeptualisiert. Dabei wird die Qualität der makro-adaptiven von der mikro-adaptiven Lernunterstützung unterschieden. Letztere wird getrennt in fachunabhängige Unterstützungsqualität (Gruppenmanagement, emotionale Wärme) und in instruktionale, mathematikbezogene Unterstützungsqualität (Lernanregung, Verwendung Fachsprache). Zweitens wird ein Messinstrument (Rating) zur Analyse der Qualität der Lernunterstützung in mathematischen Lernsituationen vorgestellt. Dieses wurde zur Analyse von videografierten Regelspielsituationen und anschließenden Leitfadeninterviews von 145 pädagogischen Fachkräften aus Deutschland und der Schweiz eingesetzt. Pro Fachkraft standen zwei Videosequenzen und zwei Interviews zur Verfügung. Auf der Basis dieser Daten wird das Messinstrument hinsichtlich verschiedener Gütekriterien sowie des Einflusses der Ausbildung der Fachkräfte (akademisch, nicht akademisch) und des Bildungskontextes (Deutschland, Schweiz) analysiert. Die theoretisch angenommene Unterscheidung von Gruppenmanagement, emotionaler Wärme und instruktionaler Unterstützungsqualität lässt sich durch eine konfirmatorische Faktorenanalyse stützen. Die Ergebnisse bestätigen die Bedeutung des Einbezugs von Planung und Reflexion als Dimensionen von adaptiver Lernunterstützungsqualität.

## Abstract (en)

Good adaptive support by educational professionals is crucial for pre-school children’s mathematical learning. Thus there is a need for appropriate instruments to evaluate the quality of the planning, implementation, and reflection process for mathematical learning opportunities in kindergarten. Existing instruments tend to focus on micro-adaptive learning support (MI-ALS), which considers the interaction between the professionals and the children. The quality of macro-adaptive learning support (MA-ALS), the teachers’ planning of and reflection on learning opportunities, has yet to be comprehensively investigated. MA-ALS is important for the acquisition of subject-related (mathematical) competence, an aspect of kindergarten education that is gaining in importance in the pre-school curricula of many countries.

The quality of mathematical learning support in kindergarten is conceptualized by differentiating between the quality of MA-ALS and MI-ALS. MI-ALS is also divided into general support (group management, emotional warmth) and subject-related support (learning stimulation, subject-specific language). A rating tool for analyzing the quality of mathematical learning support is presented and its psychometric quality is assessed. The tool is then used to analyze the teaching quality in two guided play situations (recorded) and two interviews each with 145 education professionals in Germany and Switzerland. A confirmatory factor analysis (CFA) confirms the validity of the distinction between group management, emotional warmth and subject-related support. The relationship between the level of MI-ALS and MA-ALS, the training of the teachers (academic vs non-academic), and the educational context (Germany vs Switzerland) is examined. The results corroborate the importance of including planning and reflection as a dimension of learning support quality.

## Einleitung

Kinder erwerben bereits vor dem Schuleintritt mathematische Kompetenzen, die eine wichtige Grundlage für die weitere mathematische Entwicklung bilden bzw. die sich als Prädiktoren für die späteren mathematischen Leistungen erwiesen haben (z. B. Benz et al. 2015; Lehrl et al. 2017; Manfra et al. 2017). Damit die Kinder sich optimal entwickeln können, ist sowohl das vorschulische mathematikbezogene Lernangebot (z. B. Anders et al. 2012; Lehrl et al. 2017) als auch die Qualität der Lernunterstützung durch die pädagogische Fachkraft relevant (z. B. Hardy und Steffensky 2014; Sylva et al. 2011).

Ein wichtiger Aspekt der Qualität von Lernangeboten ist deren adaptive Gestaltung, d. h. die laufende Beobachtung des Lernfortschritts, der Motivation und des Verhaltens der Kinder und ein darauf abgestimmter Unterricht (Parsons et al. 2018). Qualitativ hochwertige Lerngelegenheiten zeichnen sich durch eine hohe Adaptivität der Lernunterstützung aus, die einerseits angepasst an die Lernbedürfnisse der Lernenden geplant, andererseits in der jeweiligen Lehr-Lernsituation ad hoc geleistet werden muss. Corno und Snow (1986) unterscheiden dabei makro- und mikro-adaptive Handlungen. Makro-Adaptionen umfassen die längerfristige Planung von Lern- und Unterrichtssituationen, Mikro-Adaptionen die direkte Interaktion zwischen der Lehr- bzw. der Fachkraft und dem Kind während der Lernsituation.

Allerdings zeigt sich hinsichtlich Umsetzung der adaptiven mathematischen Lernunterstützung im Vorschulbereich bzw. im Kindergarten^1^ und ihrer Qualität Entwicklungsbedarf. Erstens weisen die wenigen Studien zur mathematikbezogenen Lernunterstützung auf ein geringes Vorkommen adaptiv gestalteter Interaktionen zwischen der pädagogischen Fachkraft und den Kindern hin (Bruns 2014; Hüttel und Rathgeb-Schnierer 2014). Zweitens wurde die Qualität der makro-adaptiven Lernunterstützung – als der Qualität der Planung der Förderung – zwar in den Studien von Bruns (2014), Sylva et al. (2006, 2018) und Wullschleger (2017) berücksichtigt, aber noch nicht systematisch untersucht. Diese Planung von Lernangeboten gewinnt jedoch im Kontext der verstärkten Arbeit mit Bildungsplänen im Kindergarten (z. B. Deutschschweizer Erziehungsdirektorenkonferenz 2014) an Bedeutung.

Weiter sind zur Erfassung der Qualität der mathematischen Lernunterstützung geeignete Instrumente notwendig. Bestehende Instrumente für die Einschätzung der adaptiven Lernunterstützung von Fachkräften im Vorschulbereich (z. B. Bruns 2014; Pianta et al. 2008; Pohle et al. 2019; Sylva et al. 2006, 2018; Wullschleger 2017) fokussieren stark auf die Ad-hoc-Lernunterstützung. Der Aspekt der Planung wird bisher nur zum Teil einbezogen. Auch diesbezüglich zeigt sich somit Entwicklungsbedarf.

In diesem Artikel wird erstens ein Instrument vorgestellt und hinsichtlich seiner Validität überprüft, mit dem ausgehend von der theoretischen Unterscheidung von makro- und mikro-adaptiver Lernunterstützung (Corno und Snow 1986; Hammond und Gibbons 2005, Schön 1983) sowohl die Qualität der Vor- und Nachbereitung von Lernsituationen als auch die Qualität der konkreten mathematischen Lernunterstützung während der Lernsituation gemessen werden soll. Es wird geprüft, ob sich die theoretisch differenzierbaren Dimensionen der Qualität der mikro- sowie der makro-adaptiven Lernunterstützung empirisch trennen lassen. Zweitens wird analysiert, ob sich das Instrument für die vergleichende Messung in zwei unterschiedlichen Bildungskontexten (Deutschland und Schweiz) eignet, indem die Messinvarianz überprüft wird. Dies ist notwendig, da zum Teil große Unterschiede zwischen den Bildungskontexten bestehen, insbesondere auch hinsichtlich der mathematischen Förderung (Gasteiger et al. 2020). Drittens wird als weiterer Validitätsaspekt untersucht, ob sich die Qualität der adaptiven Lernunterstützung nach Ausbildung der Fachkräfte (akademisch vs. nicht-akademisch) unterscheidet.

Die Untersuchung erfolgt in geplanten, regelspielbasierten mathematischen Lernsituationen mit 4–6-jährigen Kindern in Kindergärten in der Schweiz und in Deutschland. Regelspiele werden gewählt, weil das Spielen grundsätzlich als Entwicklungsmotor für die kognitive und soziale Entwicklung gilt und im Kindergartenalltag bedeutsam ist (Leuchter 2013). Zudem hat sich der Einsatz von mathematikhaltigen Regelspielen als effektive Fördermaßnahme früher mathematischer Fähigkeiten herausgestellt (z. B. Gasteiger und Moeller 2021; Jörns et al. 2014; Vogt et al. 2018). Schließlich bieten sich Regelspiele durch ihre klare Zielsetzung und Strukturierung besonders zur Lernunterstützung durch die Fachkräfte an (Weisberg et al. 2016).

## Regelspielunterstützte mathematische Förderung im Kindergarten

Die vorschulische Förderung mathematischer Kompetenzen wird heute als zentral für die weitere mathematische Entwicklung der Kinder erachtet (z. B. Hasemann und Gasteiger 2020; Benz et al. 2015). Verstärkt wird dies u. a. durch die Fokussierung auf das akademische Lernen im Kindergarten in vielen Ländern (z. B. DeLuca et al. 2020) und die damit verbundene Erstellung von entsprechenden Bildungsplänen (z. B. Deutschschweizer Erziehungsdirektorenkonferenz 2014). Als ein mögliches mathematisches Lernangebot wird vorgeschlagen, unterschiedliche Spielsituationen zur Förderung mathematischer Kompetenzen im Vorschulalter zu nutzen (Schuler 2013). Dabei können zwei Formen des Spiels unterschieden werden: das Spiel als Ziel und das Spiel als Mittel zum Lernen (Heimlich 2015). Spiele als Mittel zum Lernen werden oftmals angeleitet. Nach Pyle und Danniels (2017) zeichnet sich das angeleitete Spiel dadurch aus, dass Erwachsene in Interaktion mit den Kindern unter Einbezug der verwendeten Spielmaterialien kommentieren, mitspielen, Fragen stellen und alternative Vorgehensweisen aufzeigen oder gezielt mathematische Ziele fördern (Gasteiger und Moeller 2021; Laski und Siegler 2014; Ramani et al. 2012). In diesen Bereich fallen auch Regelspiele (Hauser 2013), die für die vorliegende Untersuchung bedeutsam sind. Empirische Ergebnisse deuten darauf hin, dass Regelspiele für die mathematische Förderung wirksam sind (z. B. Gasteiger et al. 2015; Jörns et al. 2014, 2017; Laski und Siegler 2014; Vogt et al. 2018). Allerdings scheint dafür auch die adaptive Lernunterstützung bedeutsam zu sein, beispielsweise indem die Kinder angeregt werden, die Aufmerksamkeit auf die den Spielen inhärenten mathematischen Aspekte zur richten (Weisberg et al. 2016). Doch nicht in allen Studien lässt sich ein Langzeiteffekt der regelbasierten Förderung nachweisen. Jörns et al. (2017) vermuten, dass die fehlenden Langzeiteffekte in ihrer Untersuchung auch auf einen wenig adaptiven Spieleinsatz zurückzuführen sein könnten und dass die Kinder mit Spielen konfrontiert wurden, die sie über- oder unterforderten. Das heißt, es wird auf allfällige Mängel in der makro-adaptiven Lernunterstützung hingewiesen. Ausgehend von solchen Überlegungen wird in der vorliegenden Studie die Qualität der adaptiven Lernunterstützung der Fachkräfte in geplanten, angeleiteten mathematischen Regelspielsituationen untersucht. Im Folgenden werden Grundlagen der adaptiven Lernunterstützung im Kindergarten beschrieben.

## Adaptive Lernunterstützung im Kindergarten

### Makro- und mikro-adaptive Lernunterstützung

Für den Elementarbereich umfasst Adaptivität nach Gasteiger (2014) die Planung und das Gestalten mathematischer Lernsituationen mit geeigneten Aktivitäten, das Anpassen der Lernsituationen, das Analysieren der mathematischen Entwicklung der Kinder sowie das Angebot von geeigneten Unterstützungsmaßnahmen in natürlichen Lernsituationen (z. B. Freispiel). Vereinfacht gesagt, können bei diesen Unterstützungsmaßnahmen zwei verschiedene Anforderungsbereiche unterschieden werden: Das adaptive Gestalten von Lernsituationen, das eine gezielte Planung und Kenntnisse des Entwicklungsstandes der Kinder voraussetzt sowie die adaptive Unterstützung innerhalb einer konkreten Lernsituation, die auch spontanes Handeln erfordert. Diese zwei Bereiche werden in Anlehnung an das Konzept des adaptiven Unterrichtens von Corno und Snow (1986), an Schöns (1983) Konzept der Reflexion von Lehrkräften sowie an Hammonds und Gibbons’ (2005) Verständnis von Scaffolding als makro- und mikro-adaptive Lernunterstützung bezeichnet. Die Aufgaben, die den Lehrkräften in diesen Konzepten zugewiesen werden, lassen sich auch auf pädagogische Fachkräfte im Kindergarten übertragen.

Makro-Adaptionen beziehen sich im Kontext des Kindergartens auf Aktivitäten der pädagogischen Fachkraft, die in der Vor- und Nachbereitung von Lernsituationen stattfinden und auf eine Passung des Angebots für die Lernenden abzielt. Mikro-Adaptionen dagegen fokussieren auf die direkte Interaktion zwischen der Fachkraft und dem Kind mit dem Ziel, die Lernsituation ständig an die Voraussetzungen der Lernenden anzupassen. Mikro-Adaptionen geschehen gemäß Corno und Snow (1986) oft spontan, sind also nicht so planbar wie Makro-Adaptionen.

Die Auffassung, dass qualitativ hochwertige Lernsituationen aus Anpassungsprozessen auf Makro- und Mikro-Ebene resultieren, spiegelt sich auch in Modellen zur Beschreibung von mathematikbezogenen Kompetenzen von Lehrkräften und frühpädagogischen Fachkräften. Nach Lindmeier (2011) steht adaptive Lernunterstützung in einem engen theoretischen Zusammenhang mit den reflexiven resp. aktionsbezogenen Kompetenzen von pädagogischen Fachkräften: Fachkräfte mit hoher reflexiver Kompetenz (z. B. hinsichtlich der Planung von Lernsituationen oder der Auswahl von geeigneten Aufgaben) verfügen über Dispositionen, um Lernsituationen adaptiv auf der Makro-Ebene zu planen. Fachkräfte mit hoher aktionsbezogener Kompetenz haben Dispositionen, um Kinder während des Lernprozesses adaptiv unterstützen zu können. Auch Gasteiger und Benz (2016) betonen, dass nicht nur situative, sondern auch geplante Lernprozesse wichtig sind. Sie haben ein Struktur-Prozessmodell mit den Facetten Wissen, situative Beobachtung und Wahrnehmung, pädagogisch-didaktische Handlung sowie Evaluation entworfen. Für die individuumsbezogene Diagnose und Förderung, die als adaptive Lernunterstützung interpretiert werden kann, werden bei den pädagogisch-didaktischen Handlungen der Fachkräfte – analog zum Modell von Lindmeier – geplante und spontane Handlungen unterschieden. Zur Planung gehört die „Auswahl adäquater mathematischer Lerngelegenheiten“, zum spontanen Handeln das „gezielte, förderdiagnostische Nachfragen“ (ebd., S. 280).

In der Unterrichtsforschung im deutschsprachigen Raum wird Adaptivität im Rahmenkonzept der drei Basisdimensionen (Klassenmanagement, kognitive Aktivierung, konstruktive Unterstützung; Praetorius et al. 2018) als „querliegend“ zu den Basisdimensionen von Unterrichtsqualität betrachtet (Praetorius und Charalambous 2018). Das bedeutet, dass die makro- und mikro-adaptive Lernunterstützung bezogen auf jede Basisdimension analysiert werden kann. Dieses Verständnis von Adaptivität lässt sich auch auf Konzepte zur Qualität der Lernunterstützung im frühpädagogischen Bereich (z. B. Pianta und Hamre, 2009) übertragen, da diese gemäß Hardy und Steffensky (2014) und Kuger und Kluczniok (2009) große Überschneidungen mit dem Konzept der Basisdimensionen aufweisen. Lernunterstützung wird im Folgenden mit den von Pianta und Hamre (2009) im CLASS-Beobachtungsinstrument herausgearbeiteten drei Domänen emotionale Unterstützung, classroom organisation im Sinne der Klassenführung (im Folgenden im Kontext des Kindergartens als Gruppenmanagement bezeichnet) und instruktionale Unterstützung operationalisiert. Zwei dieser Domänen sind fachunabhängig (Gruppenmanagement, emotionale Unterstützung), die instruktionale Unterstützung erfolgt in der Praxis auf einen bestimmten Lerngegenstand, z. B. Mathematik bezogen. Auch Hardy und Steffensky (2014) betonen, dass für die adaptive Lernunterstützung sowohl domänenspezifische Interaktionen, als auch Aspekte der emotionalen Unterstützung und der Strukturierung von Lernsituationen wichtig sind. In den folgenden zwei Abschnitten wird deshalb unterschieden zwischen der Qualität der fachunabhängigen und der fachbezogenen Lernunterstützung.

Ausgehend vom aktuellen Forschungsstand werden im Folgenden Merkmale einer qualitativ hochwertigen makro- und mikro-adaptiven Lernunterstützung bezogen auf die mathematische Förderung mit Regelspielen im Kindergarten dargestellt.

### Diagnostizieren, planen und reflektieren: Qualität der makro-adaptiven mathematischen Lernunterstützung im Kindergarten

In Anlehnung an die in Abschn. 3.1 dargestellten Überlegungen gehört zur makro-adaptiven Lernunterstützungsqualität im Kindergarten die Vor- und Nachbereitung einer Lernsituation, bestehend aus der Planung auf der Basis der Diagnose des Lernstandes der Kinder, aus der Reflexion der Lernsituation und aus der weiterführenden Förderplanung. Die Planung umfasst fachunabhängige Überlegungen zum Gruppenmanagement im Kindergarten (z. B. Rituale, Rhythmisierung im Tagesablauf, Raumgestaltung). Fachbezogen sind Maßnahmen zur instruktionalen Unterstützung wie z. B. die Auswahl der zum Lernstand der Kinder passenden Materialien oder Spiele oder das Vorbereiten von geeigneten Hilfsmitteln für eine wertschätzende Unterstützung individueller Lernwege. Gelungene makro-adaptive Lernunterstützung zeigt sich darin, dass Lernsituationen abgestimmt auf den Lernstand der Kinder (kontinuierliche Beobachtung und Dokumentation) geplant und nach der Durchführung reflektiert werden. Ausgehend davon werden weitere Schritte der Förderung festgelegt. Dieses qualitätssichernde Vorgehen erhält aufgrund der zunehmenden Betonung des akademischen Lernens, insbesondere im Kontext der Entwicklung von Bildungsplänen und Standards für den Kindergarten, verstärkt Bedeutung (DeLuca et al. 2020).

Empirische Befunde weisen darauf hin, dass in der Praxis Entwicklungsbedarf besteht bezüglich der Umsetzung einer qualitativ hochwertigen makro-adaptiven mathematischen Lernunterstützung (Bruns 2014; Wullschleger 2017). Bruns (2014) hat die Adaptivität der Planung bezogen auf die Festlegung von individuellen Lernzielen und die Angemessenheit von mathematischen Aktivitäten von Fachkräften in Deutschland analysiert. Nur etwa ein Drittel der Fachkräfte hat individuelle Lernziele für ausgewählte Kinder formuliert und die geplanten mathematischen Aktivitäten wichen deutlich von den vorgängig eingeschätzten Bedürfnissen der Kinder ab. Wullschleger (2017) kommt zu dem Schluss, dass es den Fachkräften in der Schweiz relativ gut gelingt, den aktuellen mathematischen Lernstand der Kinder zu erfassen, dass es ihnen jedoch schwerfällt, Kinder darauf aufbauend adaptiv bei den Lernprozessen zu unterstützen. Jörns et al. (2017) sehen im wenig adaptiven Spieleinsatz und in einer nicht optimalen Planung der mathematischen Spielsituationen Gründe für die fehlende Langzeitwirkung einer spielbasierten mathematischen Förderung in deutschen Kindergärten.

Dieser Überblick deutet einerseits darauf hin, dass hinsichtlich der Verbesserung der Qualität der makro-adaptiven mathematischen Lernunterstützung Handlungsbedarf zu bestehen scheint. Anderseits zeigt sich aber auch, dass die Qualität der makro-adaptiven Lernunterstützung erst in wenigen Studien explizit untersucht wurde.

### In der direkten Interaktion unterstützen: Qualität der mikro-adaptiven mathematischen Lernunterstützung im Kindergarten

Der Konzeptualisierung von Corno und Snow (1986) folgend bemisst sich die Qualität der mikro-adaptiven Lernunterstützung an der Qualität der konkreten Unterstützungsmaßnahmen, die eine pädagogische Fachkraft in der Interaktion mit den Kindern realisiert, einerseits fachabhängig, andererseits fachunabhängig (Abschn. 3.1).

Als fachunabhängiger Bereich ist die Qualität des Gruppenmanagements zentral. Dieses umfasst nicht nur den Umgang mit Störungen des Lernprozesses, sondern auch alle Bemühungen der pädagogischen Fachkraft, einen möglichst störungsfreien Ablauf der Lernsituation zu gewährleisten, sodass die zur Verfügung stehende Lernzeit effektiv genutzt werden kann. Zu einem gelingenden Gruppenmanagement gehört somit das Schaffen von störungsfreien Spiel- und Lernsituationen, die darauf abzielen, kognitives und soziales Lernen der Kinder zu ermöglichen (Dollase 2015; Wannack und Herger 2021).

Neben dem Gruppenmanagement stellt die emotionale Unterstützung ein fachunabhängiges Qualitätskriterium dar. Lernen findet bei (jungen) Kindern vor allem in sozialen Beziehungen statt. Der Aufbau einer tragfähigen Beziehung und die emotionale Unterstützung des einzelnen Kindes stellen eine zentrale Aufgabe der pädagogischen Fachkräfte dar (Ahnert und Gappa 2013; Koch 2013). Die Qualität der Lernunterstützung wird dementsprechend als eine den Kindern zugewandte, wertschätzende Haltung und als ein von emotionaler Wärme geprägtes Verhalten der pädagogischen Fachkraft beschrieben (Nentwig-Gesemann et al. 2011; Whitebread 2015).

Für die Qualität der fachbezogenen Lernunterstützung sind im Kontext von mathematikhaltigen Spielsituationen zwei Aspekte wichtig: das Anregen von Lernprozessen sowie die Verwendung der Fachsprache. Die Lernprozessanregung ist dann qualitativ hochwertig, wenn es der pädagogischen Fachperson gelingt, das mathematische Lernen der Kindergartenkinder verständnisorientiert und elaboriert anzuregen und zu unterstützen (z. B. Wadepohl 2015). Typische Unterstützungsstrategien sind gezielte Anregungen und Hinweise, ein produktiver Umgang mit Fehlern, herausfordernde und kognitiv anregende Fragen oder gemeinsam geteilte Denkprozesse (Wullschleger 2017). Zentral ist, dass es gelingt, diese Strategien adaptiv einzusetzen und auf den jeweiligen mathematischen Lerninhalt hin auszurichten.

Was die Verwendung der Fachsprache betrifft, haben Klibanoff et al. (2006) aufgezeigt, dass der sogenannte „math talk“, die Verwendung von mathematikbezogener Sprache, einen positiven Einfluss auf die mathematische Lernentwicklung der Kinder nimmt. Auch Hardy und Steffensky (2014) weisen auf die Bedeutung der Angemessenheit der sprachlichen Unterstützung für den Kompetenzaufbau der Kinder hin. Zudem wird der enge Zusammenhang von sprachlichen Kompetenzen und mathematischem Lernen betont (Kempert et al. 2019). Eine hohe Qualität der fachsprachlichen Anregung liegt dann vor, wenn die Kinder bei der Fachkraft die präzise Verwendung passender mathematischer Begriffe erfahren können und sie zu deren Anwendung ermuntert werden.

Zusammenfassend kann festgehalten werden, dass die mikro-adaptive Lernunterstützung fachunabhängig mit dem Gruppenmanagement und der emotionalen Unterstützung erfasst werden kann. Fachabhängig sind das Anregen von Lernprozessen und die Verwendung der Fachsprache zentral.

## Erfassung der Qualität von Lernunterstützung

Für die Erfassung der makro-adaptiven Lernunterstützung ist ein Vorgehen notwendig, mit dem die Überlegungen der Fachkräfte zur Planung und zur Reflexion erfasst werden können. Dazu eignen sich Fragebögen (Bruns 2014), Interviews (Bruns 2014; Wullschleger 2017) sowie schriftliche Planungen (Sylva et al. 2011). Für die Erfassung der Qualität mikro-adaptiver Lernunterstützung muss ein Zugang gewählt werden, der es erlaubt, die Interaktionen, Handlungen und spontanen Reaktionen in einer konkreten Situation zu erfassen. Dazu eignen sich Beobachtungen (Clements und Sarama 2008) und insbesondere Videodaten (Pohle et al. 2019; Wullschleger 2017), mittels derer die jeweiligen Situationen wiederholt analysiert werden können.

### Instrumente zur Erfassung von adaptiver (mathematischer) Lernunterstützung in Vorschuleinrichtungen

Es gibt bereits mehrere Instrumente zur Erfassung der Qualität der Lernunterstützung in Vorschuleinrichtungen, die als Grundlage für die Entwicklung des hier vorgestellten Instruments genutzt wurden. Tab. 1 gibt einen Überblick über häufig verwendete Instrumente, die allerdings unterschiedliche Zielsetzungen und Schwerpunkte haben. CLASS (Pianta et al. 2008), COEMET (Clements und Sarama 2008), ECERS‑E (Sylva et al. 2006) und ECERS‑R (Harms et al. 2005) stammen aus dem angloamerikanischen Raum und berücksichtigen den im deutschsprachigen Raum vorherrschenden Ansatz des spielbasierten Lernens im Kindergarten nur unzureichend (Pohle et al. 2019). KES‑E (Sylva et al. 2018) und KES‑R (Tietze et al. 2007) wurden ausgehend von den ECERS-Skalen für den Kontext in Deutschland adaptiert. Die Instrumente von Hüttel und Rathgeb-Schnierer (2014), Wullschleger (2017) sowie Pohle et al. (2019) wurden im Rahmen unterschiedlicher Projekte entwickelt und weisen jeweils verschiedene Bezüge zu den vorab beschriebenen Instrumenten auf.

**Table 1:** 

Instrument/Kontext	Erfasste Bereiche	Einschätzung				
		Spielbasiert	Fachspezifisch	Makro-adaptiv	Mikro-adaptiv	Domänen
CLASS, USA (Pianta et al. 2008)	Interaktion Fachkraft–Kind: emotional support, classroom organisation, instructional support	–	–	–	✔	✔
COEMET, USA (Clements und Sarama 2008)	Elemente im Klassenzimmer, Klassenzimmerkultur, spezifisch mathematische Aktivitäten	–	✔	–	✔	z. T.
ECERS‑E, UK (Sylva et al. 2006)	Domänenspezifische Aspekte Fachsprachliche Anregungen (language reasoning) Interaktionen (Fachkraft-Kind; Kind-Kind) Schriftliche Planungen und methodische Differenzierungen	–	✔	z. T.	z. T.	z. T.
Darauf aufbauend KES‑E, D (Sylva et al. 2018)		–	✔	z. T.	z. T.	z. T.
ECERS‑R, UK (Harms et al. 2005)	Ausstattung, Betreuung, Anregung, Aktivitäten, Interaktionen, Strukturierung	–	–	z. T.	z. T.	z. T.
Darauf aufbauend KES‑R, D (Tietze et al. 2007) KES-RZ, D (Nattefort und Grenner 2017)		–	–	z. T.	z. T.	z. T.
PRIMEL, D (Hüttel und Rathgeb-Schnierer 2014)	Lernprozessgestaltung, Emotionsregulation/Beziehungsgestaltung, Klassenführung, kogn. Aktivierung: Mathematik	✔	✔	–	✔	✔
Wullschleger (2017), CH	Qualität der Lernstandsdiagnose, Adaptivität der Lernunterstützung	✔	✔	✔	✔	z. T.
MiA-Num, D (Pohle et al. 2019)	Klarheit des mathematischen Inhalts, kognitive Aktivierung, konstruktive Lernunterstützung	✔	✔	–	✔	z. T.

Der Überblick in Tab. 1 zeigt, dass die wenigsten Instrumente aus dem angloamerikanischen Raum für eine fachlich-mathematische Bewertung der Lernunterstützung geeignet sind. Mit drei Instrumenten lässt sich die Qualität der makro-adaptiven Lernunterstützung ganz oder teilweise einschätzen. Die drei Domänen Gruppenmanagement, emotionale und instruktionale Unterstützung werden in den Instrumenten ebenfalls unterschiedlich berücksichtigt. Es zeigt sich somit, dass ein Instrument fehlt, mit dem die fachspezifische mikro- und makro-adaptive Lernunterstützung in den Domänen Gruppenmanagement, emotionale Unterstützung und fachliche Unterstützung in regelspielbasierten Situationen erfasst werden kann.

Die Übersicht zeigt, dass ein Instrument fehlt, das sich für die Analyse der fachlich-mathematischen Lernunterstützung im spezifisch regelspielorientierten Kontext des Kindergartens eignet und sowohl die makro-adaptive als auch die mikro-adaptive Lernunterstützung und die drei genannten Domänen berücksichtigt. Ein solches Instrument sollte zudem nicht zu abhängig von einem kulturellen Kindergartenkontext sein und die Qualität der Lernunterstützung in unterschiedlichen Referenzrahmen wie beispielsweise in Deutschland und der Schweiz vergleichbar erfassen können.

### Berücksichtigung von Kontextvariablen und Messinvarianz

Wenn ein Instrument in unterschiedlichen Kontexten wie in dieser Untersuchung in zwei Ländern eingesetzt wird, dann sind entsprechende Kontextvariablen zu berücksichtigen. In der frühkindlichen Bildung und in der Ausbildung der Fachkräfte gibt es zwischen Deutschland und der Schweiz einige Unterschiede, die berücksichtigt werden müssen (Gasteiger et al. 2020). So gehört der Kindergarten in der Schweiz im Gegensatz zu Deutschland zur Volksschule und ist entsprechend unentgeltlich. Zudem sind in den nationalen Bildungsstandards^2^ und im Lehrplan 21 (Deutschschweizer Erziehungsdirektorenkonferenz 2014) für den Kindergarten verbindliche fachliche Kompetenzen für das mathematische Lernen formuliert. Dieser hat somit einen klaren Bildungsauftrag. Damit verbunden ist ein sozialkonstruktivistisches Verständnis von Lernen, bei dem die Fachkräfte und die Kinder als Ko-Konstrukteure von Wissen und Kultur in der Zone der nächsten Entwicklung des Kindes interagieren (Fthenakis 2002; Vygotsky 1978).

In Deutschland hat der Kindergarten unterschiedliche Trägerschaften und die Eltern bezahlen Beiträge (Gasteiger et al. 2020). Auch in Deutschland gibt es Bildungs- und Orientierungspläne. Diese unterscheiden sich jedoch inhaltlich beträchtlich von Bundesland zu Bundesland und in einigen Fällen wird die mathematische Förderung nur am Rande erwähnt (Gasteiger et al. 2020). Die Bildungspläne basieren auf dem Situationsansatz, der das Lernen junger Kinder in sozialen Situationen betont und von den alltäglichen Erfahrungen und Interessen der Kinder ausgeht (Anders 2015).

Unterschiede zwischen den beiden Ländern zeigen sich auch in der Ausbildung. In der Schweiz ist diese seit 2001 auf tertiärer Stufe angesiedelt (Schweizerische Konferenz der kantonalen Erziehungsdirektoren 2007). In Deutschland werden Erzieher*innen meistens an Fachschulen für Sozialpädagogik ausgebildet, die dem Sekundar- bzw. Postsekundarbereich zugeteilt sind (Oberhuemer et al. 2010). Die Thematik der frühen mathematischen Förderung wird wenig spezifisch behandelt. Vor diesem Hintergrund wird erwartet, dass Fachkräfte mit einer akademischen Ausbildung und Fachkräfte aus dem bildungsorientierten Kontext der Schweiz eine höhere Qualität der adaptiven Lernunterstützung aufweisen als nicht-akademisch ausgebildete Fachkräfte bzw. als Fachkräfte aus Deutschland.

Da das Instrument in Deutschland und der (deutschsprachigen) Schweiz einsetzbar sein soll, muss die Messinvarianz des Instruments überprüft werden. Sie ist eine notwendige Bedingung, um valide Vergleiche zwischen Gruppen zu ermöglichen (Chen 2008; Cieciuch et al. 2016) und sicherzustellen, dass die Skalen in beiden Gruppen dasselbe messen bzw. gleich funktionieren (Schwab und Helm 2015).

Schließlich sind Strukturmerkmale der Kindergartengruppen zu berücksichtigen, wenn die Qualität der Lernunterstützung erfasst werden soll. Kuger und Kluczniok (2009) haben in ihrer Studie festgestellt, dass sich das Alter der Kinder auf die Qualität der Gruppenführung, das Klima und die mathematische Förderung auswirkte. Zudem sank die Prozessqualität signifikant mit zunehmendem Anteil von Kindern mit Migrationshintergrund in einer Gruppe.

Sowohl die Kontextfaktoren Land und Ausbildung als auch die in dieser Studie erhobenen Stichprobenmerkmale Alter und Erstsprache der Kinder müssen bei den Analysen berücksichtigt werden, falls sich Unterschiede zwischen den Länderstichproben zeigen.

## Zielsetzungen der Studie

Ausgehend von den erläuterten theoretischen Annahmen, den empirischen Befunden zur adaptiven Lernunterstützung und den bestehenden Instrumenten hat diese Querschnittstudie das Ziel verfolgt, ein Instrument zur Erfassung der Qualität von mathematikbezogener adaptiver Lernunterstützung zu entwickeln und dessen Güte zu überprüfen. Das Instrument soll Aspekte aufnehmen, die in bestehenden Instrumenten (Tab. 1) fehlen oder nur teilweise berücksichtigt werden. Es soll für den regelspielorientierten Kontext des Kindergartens geeignet sein, die makro- und die mikro-adaptive Lernunterstützung sowie die emotionale Unterstützung, das Gruppenmanagement und die fachliche Lernunterstützung berücksichtigen, in unterschiedlichen nationalen Kontexten (Deutschland, Schweiz) vergleichend einsetzbar sein und eine geeignete Sensitivität für Qualitätsunterschiede aufweisen.

Vor dem Hintergrund dieser Ziele interessiert nach einer theoriegeleiteten Entwicklung erstens, ob die Qualität der mathematikbezogenen adaptiven Lernunterstützung durch das Instrument reliabel erfasst werden kann. Zweitens wird untersucht, ob sich die theoretisch angenommene Unterscheidung in makro- und mikro-adaptive Lernunterstützungsqualität in den Daten bestätigen lässt. Drittens wird die Messinvarianz des Instruments für Substichproben aus Deutschland und aus der Schweiz überprüft, da Skalen für unterschiedliche Gruppen nicht immer gleich gut funktionieren (z. B. Nusser et al. 2015). Viertens wird im Hinblick auf die Sensitivität analysiert, ob sich Fachkräfte mit und ohne akademische Ausbildung sowie Fachkräfte aus der Schweiz und aus Deutschland hinsichtlich der Qualität der adaptiven Lernunterstützung unterscheiden (Known-Group-Kontraste). Bezüglich der makro-adaptiven Lernunterstützungsqualität sowie der fachlichen mikro-adaptiven Lernunterstützungsqualität wird erwartet, dass Fachkräfte mit einem akademischen Abschluss höhere Werte erreichen, da die frühe mathematische Bildung im Rahmen dieser Ausbildung spezifischer thematisiert wird. Dasselbe wird auch für Schweizer Fachkräfte erwartet, da der Kindergarten dort stärker bildungsorientiert und teils sogar schulisch organisiert ist. Dagegen wird bei der fachunabhängigen mikro-adaptiven Lernunterstützungsqualität (emotionale Wärme, Gruppenmanagement) angenommen, dass es keine Unterschiede zwischen den Gruppen gibt, da weder die akademische Ausbildung noch die Bildungsorientierung für diese grundlegenden Qualitätsmerkmale eine entscheidende Rolle spielen sollten. Ausgehend von den Ergebnissen von Kuger und Kluczniok (2009) wird fünftens überprüft, ob sich die Stichprobenmerkmale Alter und Erstsprache auf die Qualität der adaptiven Lernunterstützung auswirken.

## Methode

### Vorgehen

Die Daten der Studie stammen aus 68 Kindergartengruppen der Deutschschweiz sowie 77 Kindergartengruppen aus Deutschland (Meier-Wyder 2020). Pro Kindergartengruppe nahmen eine pädagogische Fachkraft sowie einige der von ihr betreuten Kinder an der Studie teil.

Um eine Vergleichbarkeit der analysierten Fördersequenzen zu erreichen, wurde ein Setting mit einem mathematikhaltigen Regelspiel gewählt, das einerseits die Interaktion zwischen der pädagogischen Fachkraft und den Kindern und andererseits auch eine Standardisierung der Spielsituation ermöglichte. Für die Qualitätseinschätzung der mikro-adaptiven Lernunterstützung wurden in jeder Kindergartengruppe zwei ca. 15-minütige Spielsituationen auf Video aufgenommen. Dazu wurde die Kamera so positioniert, dass die frontale Sicht sowohl die Kinder als auch die Fachkraft und die Spielhandlung erfasste. Die pädagogische Fachkraft spielte jeweils mit einer Kleingruppe das Würfelspiel „Goldstückspiel“ (Schmassmann und Moser Opitz 2007), mit dem verschiedene mathematische Kompetenzen gefördert werden können: Vorwärtszählen, Bestimmen einer Anzahl durch Zählen oder „auf einen Blick“, Eins-zu-Eins-Zuordnung, Zahlen lesen, Zahlen zusammensetzen, Differenz zwischen Mengen und Zahlen bestimmen, usw. Das Spiel wurde den Fachkräften im Vorfeld ausgehändigt und sie konnten sich damit in ihrem Kindergartenalltag vertraut machen. Sie erhielten zudem vor den Videoaufnahmen den Auftrag, zwei Kleingruppen von jeweils drei Kindern zu bilden, für die sie das Spiel als besonders geeignet zur mathematischen Förderung betrachteten. Sie wurden ebenfalls aufgefordert, die Kinder bei der Durchführung des Spiels anzuleiten, konkrete mathematikbezogene Hilfestellungen zu geben und eine mathematische Lernsituation zu gestalten.

Für die Erhebung von Daten zur Qualität der makro-adaptiven Lernunterstützung ist es wichtig, dass situationsgebundene Aspekte erfasst werden (vergl. Abschn. 3.1). Um dies zu gewährleisten, wurden die pädagogischen Fachkräfte direkt im Anschluss an die Videoaufnahmen mittels eines leitfadengestützten Interviews zur Planung der Spielsituation, zur Lernunterstützung sowie zur weiteren Förderplanung befragt. Die Interviews wurden mit der Videokamera aufgenommen. Die Kamera wurde gegen eine Wand gerichtet, sodass die Fachkräfte nicht im Bild erschienen und sie sich möglichst frei äussern konnten. Auf die Erfassung von schriftlichen Planungsunterlagen wurde verzichtet, da davon ausgegangen werden musste, dass nicht alle pädagogischen Fachkräfte in ihrem Arbeitsalltag schriftliche Planungen erstellen.

### Stichprobe

Insgesamt nahmen *N* = 145 frühpädagogische Fachkräfte (im Folgenden als Fachkräfte bezeichnet) aus der deutschsprachigen Schweiz (*n* = 68) sowie Deutschland (*n* = 77) freiwillig an der Untersuchung teil. Das Dienstalter betrug in der Schweiz *M* = 12,37 (*SD* = 9,36) und in Deutschland *M* = 13,26 (*SD* = 17,34) Jahre. Der Anteil der Fachkräfte in Deutschland mit einem nicht-akademischen Abschluss betrug 87 %, derjenige mit akademischem Abschluss^3^ 13 %. In der Schweiz waren die Anteile ausgeglichen (53 % akademisch, 47 % nicht-akademisch; Tab. 2)^4^.

**Table 2:** 

	Abschluss	Männlich	Weiblich	Total	Gesamt
		*n*	*n*	*n* (%)	%
		7	138	145	100
Schweiz	Nicht-akademisch	0	36	36 (52,9)	24,8
	Akademisch	0	32	32 (47,1)	22,1
	Total	0	68	68	46,9
Deutschland	Nicht-akademisch	7	60	67 (87,0)	46,2
	Akademisch	0	10	10 (13,0)	6,9
	Total	7	70	77	53,1

Die betreuten Kinder waren im Durchschnitt 5,2 Jahre alt (Schweiz 5,25 Jahre, Deutschland 5,12 Jahre). Zwischen der Schweiz und Deutschland zeigten sich signifikante Unterschiede (F [1, 889] = 24,62, *p* = 0,000, *n* = 891). Das Alter wird deshalb beim Known-Group-Vergleich berücksichtigt. Hinsichtlich der Erstsprache Deutsch (Deutschland *n* = 344, Schweiz *n* = 523) zeigten sich keine signifikanten Unterschiede zwischen den beiden Ländern (𝒳^2^ (*df* = 1) = 3,49, *p* = 0,062, *n* = 867).

### Messinstrument

Auf der Basis der erarbeiteten theoretischen Grundlagen (Abschn. 3) und in Anlehnung an bereits bestehende Instrumente (Abschn. 4.1) wurde die Qualität der makro- und mikro-adaptiven Lernunterstützung mit insgesamt 8 Items mit einer vierstufigen Skala erfasst. Eine 4 bedeutete eine volle Übereinstimmung des Unterstützungsverhaltens der Fachkraft mit den Indikatoren des jeweiligen Items (vollumfänglich beobachtbar in den Videos oder vollumfänglich den Aussagen im Interview zu entnehmen). Eine 3 bedeutete eine gute Übereinstimmung mit den Indikatoren der jeweiligen Items (mehrheitlich beobachtbar in den Videos resp. mehrheitlich den Aussagen im Interview zu entnehmen), eine 2 eine geringe Übereinstimmung und eine 1 schließlich keine Übereinstimmung.

#### Qualitätseinschätzung der makro-adaptiven Lernunterstützung

Die Erfassung der Qualität der makro-adaptiven Lernunterstützung erfolgte fachabhängig mit der Auswertung der standardisierten, leitfadengestützten Interviews (Kiker und Motowidlo 1998). Ausgehend von den Ausführungen in Abschn. 3.1 wurde die Qualität der makro-adaptiven Lernunterstützung anhand der Aussagen der Fachkräfte im Interview mit vier Items operationalisiert: Planung der Spielsituation, Lernstandsdiagnose, Reflexion der Lernunterstützung sowie Förderplanung. Jedem dieser vier Items waren spezifische Fragen im Leitfaden des Interviews zugeordnet. Für jedes dieser Items wurden theorie- und datengeleitet Indikatoren formuliert (Tab. 3) sowie Ankerbeispiele aus den Antworten der Fachkräfte im Interview notiert (Meier-Wyder 2020). Der Fokus lag bei allen Items auf der fachlichen Lernunterstützung. Auf das Erheben der Planung und Reflexion der fachunabhängigen makro-adaptiven Lernunterstützung musste aus forschungsökonomischen Gründen verzichtet werden.

**Table 3:** 

Item	Indikatoren
Planung der Spielsituation	Wissen aus der formativen Lernstandsdiagnose wird zielgerichtet zur Planung der Spielsituation verwendet. Beispielfragen: Aus welchen Gründen haben Sie diese Kinder für die beiden Spieleinheiten ausgewählt? Gab es einen bestimmten Grund für die Gruppenzusammensetzung?
Lernstandsdiagnose	Äußerungen Fachkraft zum Lernstand einzelner Kinder geben Aufschluss, dass sie Einblick in den aktuellen Lernstand des Kindes besitzt. Beispielfrage: Welches waren Ihre Erwartungen im Zusammenhang mit den mathematischen Kompetenzen der Kinder beim Spiel?
Reflexion der Lernunterstützung	Ablauf und Zielsetzung der Spielsituation werden kritisch reflektiert. Beispielfragen: An welchen Stellen war es besonders wichtig, dass Sie die Kinder begleitet haben? Gab es Momente bei der Spielbegleitung, die aus mathematischer Sicht für die Kinder besonders lehrreich waren?
Förderplanung	Aus der Reflexion werden Folgerungen für die nächste Fördersequenz gezogen. Beispielfrage: Was ist aus Ihrer Sicht wichtig für die weitere mathematische Förderung dieser Kinder?

#### Qualitätseinschätzung der mikro-adaptiven Lernunterstützung

Die Qualität der mikro-adaptiven Lernunterstützung wurde mittels eines Ratings von Videodaten aus den zwei Spielsituationen erhoben (Meier-Wyder 2020). Um die Einschätzung präzise vornehmen zu können (Merkleistung der Raterinnen und Rater) und um Veränderungen in der Lernunterstützung berücksichtigen zu können, wurde mit einem Time-Sampling gearbeitet. Die Dauer der Spielsituation (ca. 15 min) wurde gedrittelt und nach jeder Sequenz (ca. 5 min) wurde für jedes Item eine Einschätzung vorgenommen. Damit standen von jeder Fachkraft aus den zwei Spielsituationen jeweils sechs Werte pro Item zur Verfügung. Aus diesen wurde für die Berechnung der Interrater-Reliabilität ein Mittelwert gebildet. Dieses Vorgehen kann als mittel-inferentes Rating angesehen werden.

Es wurden je zwei fachunabhängige und zwei fachbezogene Items formuliert (Tab. 4). Fachunabhängig sind die Items „Emotionale Wärme“ sowie „Gruppenmanagement“. Fachbezogene Items sind die „Lernprozessanregung“ sowie die „Fachsprachliche Anregung“, diese sind spezifisch auf das mathematische Lernen bezogen (Beispiele in Tab. 4).

**Table 4:** 

Item		Indikatoren
Fachunabhängig	Emotionale Wärme	Nonverbale emotionale Wärme wird durch wohlwollende Gestik/Mimik, mit direktem Blickkontakt und Aufmerksamkeit erzeugt
		Verbale emotionale Wärme wird durch freundliche, ermutigende Sprache erzeugt. Beispiele: Die Fachkraft hat Blickkontakt, ist aufmunternd und setzt zustimmendes Nicken und ermutigende Blicke ein. Die Fachkraft lässt die Kinder in Ruhe ausreden
	Gruppenmanagement	Reibungsloser Spielablauf wird gewährleistet, um Time-on-task hoch zu halten
		Motivation wird durch Lenkung der Aufmerksamkeit aufrechterhalten
		Gegen Störungen wird vorgebeugt resp. bei Störungen wird angemessen reguliert. Beispiele: Die Fachkraft reagiert angemessen auf Störungen. Die Spielregeln werden thematisiert und auf deren Einhaltung wird geachtet
Fachlich	Mathematische Lernprozessanregung	Didaktische Handlungen der pädagogischen Fachkraft werden den mathematischen Handlungen/Äußerungen der Kinder angepasst
		Durch gezielte Erklärungen oder Hinweise, herausfordernde Fragen, produktiven Umgang mit Fehlern und strukturierte Lernprozesse wird die mathematische Kompetenz in der Zone der nächsten Entwicklung gefördert. Beispielfragen: Welche Zahl musst du würfeln, damit du auf das Feld mit den Goldstücken kommst? Wie kannst du möglichst schnell bestimmen, wie viele Punkte auf den zwei Würfeln sind?
	Fachsprachliche Anregung	Mathematische Begriffe werden von der pädagogischen Fachkraft korrekt verwendet
		Kinder werden zur Nutzung von Fachbegriffen ermuntert. Beispielfragen: Wie viele Goldstücke hast du mehr als/weniger als Kind X? Welche Zahl hast du gewürfelt? Auf welcher Zahl steht deine Spielfigur?

### Analysen

Die Qualitätsratings erfolgten anhand eines Manuals durch vier Raterinnen (alle weiblich) aus Deutschland und der Schweiz. Die Raterinnen wurden gemeinsam geschult, um den Einfluss von nationalen/kulturellen Referenzrahmen zu minimieren. Jedes Item wurde auf der oben erwähnten Skala von 1 bis 4 eingeschätzt (niedrige bis hohe Qualität). Die Prüfung der Interrater-Reliabilität erfolgte zu drei Zeitpunkten während der Phase der Analysen (nach der Analyse der ersten Videos, nach der Analyse der Hälfte der Videos und nach der Analyse aller Videos). Zur Überprüfung der Interrater-Reliabilität wurden 15 % der Daten (Videoaufzeichnungen und Interviews, die Hälfte der Daten aus der Schweiz und die andere Hälfte aus Deutschland) von allen Raterinnen eingeschätzt. Da es sich um ein intervallskaliertes Ratingsystem handelt, wurden zur Prüfung der Interrater-Reliabilität Generalisierbarkeitskoeffizienten herangezogen. Es wurden absolute und relative G‑Koeffizienten unterschieden. Der absolute Wert ist entscheidend, wenn sowohl die Rangreihe als auch die absolute Höhe des Urteils berücksichtigt werden. Beim relativen Wert ist nur die Rangreihe relevant. Das Berichten des absoluten Wertes ist dann bedeutsam, wenn nur ein Teil des Materials von allen Ratenden eingeschätzt wird. Dadurch wird sichergestellt, dass die Werte auch in der absoluten Höhe vergleichbar sind und nicht eine Person deutlich anders als die anderen urteilt (Lotz et al. 2013). Der G‑Koeffizient wurde mit dem EduG-Programm berechnet (Swiss Society for Research in Education Working Group 2010).

Um zu prüfen, ob die Daten die theoretisch angenommene Struktur abbilden, wurde eine Konfirmatorische Faktorenanalyse (CFA) durchgeführt. Die Trennung in mikro- und makro-adaptive Lernunterstützung legt einerseits ein 2‑Faktorenmodell nahe. Andererseits kann aufgrund der Aufteilung der mikro-adaptiven Lernunterstützung in einen fachbezogenen und einen fachunabhängigen Bereich ein 3‑Faktoren-Modell als plausibel angenommen werden. Entsprechend wurde in einem ersten Schritt ein 2‑Faktorenmodell geprüft und mit einem 3‑Faktorenmodell verglichen. Die statistischen Analysen wurden mit dem Statistikpaket lavaan (Rosseel 2017) in R durchgeführt. Die Faktorenladungen wurden frei geschätzt und standardisiert. Für die Beurteilung der Güte der CFA wurden neben dem χ^2^-Test die üblichen Fit-Statistiken herangezogen (Weiber und Mühlhaus 2014). Da bei zwei Items (Lernprozessanregung sowie Planung der Spielsituation) keine Normalverteilung vorlag, wurde der Maximum Likelihood Robust-Schätzer (MLR-Schätzer) verwendet. Zudem wurde eine Fehlerkorrelation für die Items „Planung der Spielsituation“ und „Lernstandsdiagnose“ zugelassen, da diese beiden Items eng zusammenhängen und im Interview häufig gemeinsam thematisiert wurden. Gemäß Brown (2015) sind solche theoriegestützten Korrekturen zulässig.

Die zweite Zielsetzung bezieht sich auf die Überprüfung der Messinvarianz. Es werden mehrere, unterschiedlich restriktive Arten von Messinvarianz unterschieden (Cieciuch et al. 2016), die in hierarchischer Beziehung stehen: konfigurale, metrische, skalare, messfehlerbezogene (strikte) und vollständige Messinvarianz. Diese werden nach der Restriktivität der Annahmen über die Gleichheit von Modellparametern zwischen den untersuchten Gruppen bestimmt. Die weniger einschränkenden Formen der Messinvarianz sind Voraussetzung für die restriktiveren Formen. Beispielsweise ist die konfigurale Messinvarianz Voraussetzung für die metrische Invarianz; diese wiederum ist Voraussetzung für die skalare Messinvarianz. Bei der konfiguralen Messinvarianz kann in beiden Gruppen (Fachkräfte CH und DE) das gleiche Modell mit den gleichen Parametern geschätzt werden, diese dürfen jedoch unterschiedliche Werte annehmen (Schwab und Helm 2015). Bei der metrischen Messinvarianz werden auch die unstandardisierten Ladungen der manifesten Variablen über die Gruppen hinweg gleichgesetzt. Sowohl die Faktorenstruktur als auch die Faktorenladungen werden als äquivalent angenommen, und es kann davon ausgegangen werden, dass die Qualität der adaptiven Lernunterstützung in beiden Ländergruppen dieselbe inhaltliche Bedeutung hat (ebd.). Bei der skalaren Messinvarianz sind auch die Intercepts der manifesten Variablen über die Gruppen hinweg identisch und für die Stichproben (Fachkräfte CH und DE) müssen invariante Faktorenstrukturen, invariante Faktorenladungen und auch invariante lntercepts (Regressionskonstanten) für die manifesten Variablen gegeben sein. Wenn die skalare Messinvarianz vorhanden ist, kann davon ausgegangen werden, dass keine itemspezifischen Schwierigkeitsunterschiede zwischen den Gruppen bestehen und die Ausprägungen der latenten Variablen zwischen den Gruppen verglichen werden können (ebd.).

Bei der Berechnung der Messinvarianz wurde der Step-Up-Ansatz verwendet. Dabei wurde mit der am wenigsten restriktiven Form der Messinvarianz (konfigurale Invarianz) begonnen, bei der mit den gleichen Parametern geschätzt wird, diese aber in den Gruppen unterschiedliche Werte annehmen dürfen (Schwab und Helm 2015). Zur Prüfung der metrischen Invarianz wurden zusätzlich die unstandardisierten Ladungen der manifesten Variablen über die Gruppen hinweg gleichgesetzt. Sowohl die Faktorenstruktur als auch die Faktorenladungen wurden als äquivalent angenommen (ebd.). Die Überprüfung, ob dieses restriktivere, genestete Modell der metrischen Invarianz passt, erfolgte mittels χ^2^-Differenztest (Christ und Schlüter 2012). Solange der Comparative Fit Index (CFI) nicht um mehr als 0,02 Einheiten sinkt und der Root Mean Square Error of Approximation (RMSEA) nicht um mehr als 0,015 Einheiten steigt, bilden beide Modelle die Datenstruktur gleich gut ab und das sparsamere Modell – das heißt das Modell, das mit weniger Parametern auskommt – wird bevorzugt (Chen 2008). Schließlich wurde mit der skalaren Invarianz geprüft, ob neben der Faktorladung auch die Achsenabschnitte über die Gruppen hinweg identisch sind. Trifft dies zu, kann davon ausgegangen werden, dass keine gruppenspezifischen Unterschiede vorliegen.

Für den Known-Group-Vergleich hinsichtlich Land und Ausbildung wurden schließlich hierarchisch-sequenzielle Regressionsanalysen durchgeführt. Bei diesem Vorgehen wird nach jedem Schritt mit einem Regressionsmodell berechnet, welcher zusätzliche Anteil der Varianz durch die Aufnahme der neuen Prädiktoren erklärt wird. Wichtig bei diesem Vorgehen ist eine analytische oder theoretisch sinnvolle Reihenfolge der einzelnen Regressionsschritte (Urban und Mayerl 2018). Da es zum Zusammenhang zwischen Land und Ausbildung noch wenige Erkenntnisse gibt und eine Festlegung der Reihenfolge nicht eindeutig ist, werden jeweils zwei Modelle mit einer unterschiedlichen Reihenfolge der Variablen berechnet.

## Ergebnisse

### Testgütekriterien und Interrater-Reliabilität

Die Reliabilität der acht Items für die Bereiche der makro- und mikro-adaptiven Lernunterstützung ergaben G‑Koeffizienten zwischen 0,78 und 0,91 (Tab. 5), was guten bis sehr guten Werten entspricht. Mit der *Varianzkomponente Rater* (VK Rater) wird aufgezeigt, wie hoch der relative Anteil der merkmalsunabhängigen Varianz ist, der durch die Unterschiede in den Einschätzungen der Videos durch die Raterinnen zustande gekommen ist. Dieser Wert entspricht der systematischen Fehlervarianz der klassischen Testtheorie und sollte demzufolge möglichst gering sein (Clausen et al. 2003). Die Spalte *Varianzkomponente Video/Interview *(VK Video bzw. Interview) bringt zum Ausdruck, wie hoch der Anteil der Varianz ist, der auf die tatsächlichen Unterschiede zwischen den Video- und Interviewsequenzen zurückzuführen ist. Aus Tab. 5 wird ersichtlich, dass die Varianzkomponente Video/Interview bei allen Merkmalen höher ist als die systematischen Unterschiede zwischen den Raterinnen (VK Rater). Tab. 5 ist auch zu entnehmen, dass sich die Raterinnen bei allen Items in ihrer Einschätzung etwas voneinander unterscheiden. Der größte Teil der Unterschiede zwischen den vergebenen Werten geht jedoch auf die Videos zurück, was den tatsächlich vorhandenen Qualitätsunterschieden der videografierten Situationen zugeschrieben wird (Meier-Wyder 2020).

**Table 5:** 

Ratingitems	Varianzkomponenten (VK)			Generalisierbarkeitskoeffizienten (G)
	Video bzw. Interview %	Rater %	VxR + *e* %	Absolut
Emotionale Wärme	60,0	4,4	35,6	0,87
Gruppenmanagement & Motivationsunterstützung	49,4	3,4	47,3	0,80
Lernprozesse adaptiv anregen	72,7	13,0	14,3	0,91
Fachsprachliche Anregung	70,5	10,2	19,3	0,91
Planung der Spielsituation	60,9	3,1	36,0	0,86
Lernstandsdiagnose	68,3	0,3	31,4	0,90
Reflexion der Lernunterstützung	50,4	2,9	46,6	0,80
Förderplanung/Ausblick	47,5	8,5	43,9	0,78

Jedes Item wurde auf einer Skala von 1 bis 4 eingeschätzt (niedrige bis hohe Qualität). Bei den Merkmalen „Lernprozess adaptiv anregen“ und „fachsprachliche Anregung“ zeigen sich zwischen den vier Raterinnen die stärksten systematischen Unterschiede (VK Rater). Die Gesamtreliabilität liegt bei α = 0,79 (Tab. 6). Dies kann bei hoch inferenten Messinstrumenten, wie es hier der Fall ist, als guter Wert angenommen werden, da die Werte bei solchen Instrumenten oft niedriger sind als bei niedrigeren inferenten Messinstrumenten (z. B. Clausen et al. 2003). Für die makro-adaptive Lernunterstützung liegt diese bei α = 0,66, was als knapp nicht zufriedenstellend aber noch akzeptabel zu bewerten ist. Da die Skala der fachlichen und der fachunabhängigen mikro-adaptiven Lernunterstützung nur aus zwei Items bestand, wurde der Spearman-Brown-Koeffizient berechnet. Dieser wies gute bzw. befriedigende Werte auf (fachunabhängig 0,78, fachlich 0,80).

**Table 6:** 

Skala	Anzahl Items	Mittelwert^a^	Std.abw	Reliabilität (Cronbachs α)	Trennschärfe
	*n*	*M*	*SD*	α	*rit*
Alle Items	8	2,70	0,52	0,79	0,39–0,67
Mikro-adaptive Lernunterstützung	4	2,61	0,50	0,83	0,58–0,77
Makro-adaptive Lernunterstützung	4	2,36	0,57	0,66	0,35–0,49

^a^1 = Qualitätsindikatoren nicht beobachtbar, 4 = Qualitätsindikatoren vollumfänglich beobachtbar

### Ergebnisse zur Überprüfung der theoretisch angenommenen Struktur

**Figure 1:**
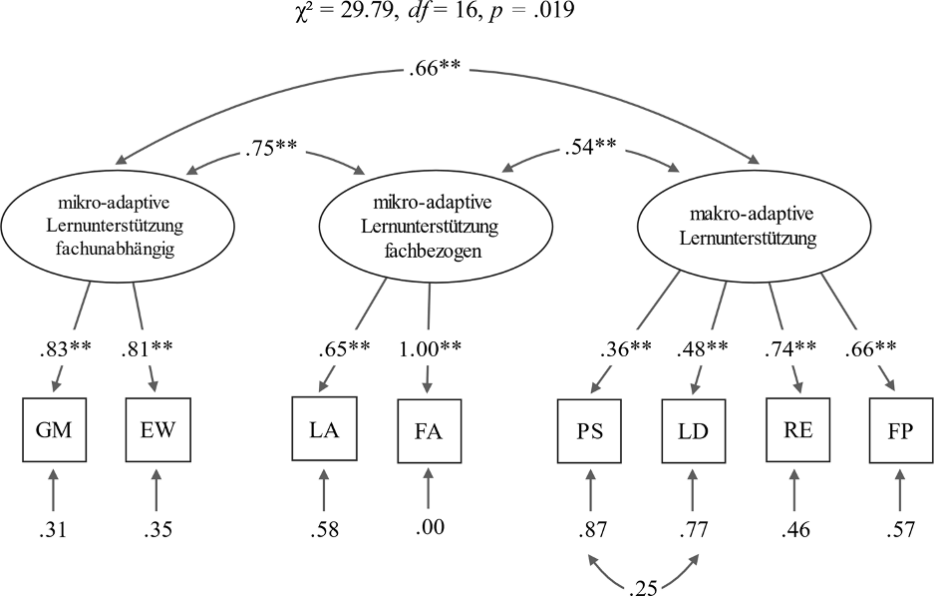

Der Vergleich eines einfaktoriellen Modells, bei dem alle Items auf einen Faktor geladen wurden (χ^2^ = 96,25, *df* = 20, *p* < 0,001, χ^2^/*df* = 4,81), mit einem 2‑Faktorenmodell (mikro- und makro-adaptive Lernunterstützung; χ^2^ = 61,63, *df* = 19, *p* < 0,001, χ^2^/*df* = 3,24) und einem 3‑Faktorenmodell (Abb. 1) ergab, dass das dreifaktorielle Modell die Struktur der vorliegenden Daten am besten abbildet (χ^2^ = 29,79, *df* = 16, *p* = 0,019, χ^2^/*df* = 1,86; Meier-Wyder 2020). Die theoretisch angenommene Unterscheidung von makro- und mikro-adaptiver Lernunterstützung sowie die Trennung der mikro-adaptiven Unterstützung in eine fachbezogene und eine fachunabhängige Dimension (3-Faktorenmodell) bestätigt sich in den CFA-Analysen und das vorliegende Modell passt zu den Daten, wie die akzeptablen (RMSEA = 0,079) resp. guten (CFI = 0,96 und SRMR = 0,052) Modellfitindizes nahelegen (Weiber und Mühlhaus 2014). Beim Item „Fachsprachliche Anregung (FA)“ beträgt der Wert ungerundet 0,998 und verweist darauf, dass der Messfehler sehr klein ist. Es wird angenommen, dass dies eine Folge davon ist, dass der Faktor nur mit zwei Indikatoren gebildet worden ist.

### Ergebnisse zur Messinvarianz

Die Ergebnisse der Messinvarianzanalysen (Tab. 7) weisen darauf hin, dass die konfigurale Invarianz für das Modell gegeben ist und das Instrument in den beiden Ländern die drei latenten Qualitätskonstrukte auf ähnliche Weise misst (*χ*^2^ = 44,31, *df* = 32, *p* = 0,07, CFI = 0,97, RMSEA = 0,065). Auch das Modell der metrischen Invarianz verschlechterte die Modellpassung nicht bedeutsam (∆CFI ≤ 0,02, ∆RMSEA ≤ 0,015), sodass metrische Invarianz vorliegt (*χ*^2^ = 45,96, *df* = 37, *p* = 0,15, CFI = 0,97, RMSEA = 0,056). Werden für das restriktivere Modell der skalaren Invarianz zusätzlich auch die Ladungen der Intercepts aller Indikatoren über die Gruppen gleichgesetzt, verändert sich der CFI um mehr als 0,02. Skalare Invarianz ist also nicht gegeben (Tab. 7).

**Table 7:** 

Modell	χ^*2*^	*p*	*df*	*χ2/df*	CFI	RMSEA	∆CFI	∆RMSEA
Konfigurale Invarianz	44,31	0,07	32	1,38	0,97	0,065	–	–
Metrische Invarianz	45,96	0,15	37	1,24	0,97	0,056	0,00	0,09
Skalare Invarianz	98,95	0,00	42	2,36	0,83	0,131	0,14	0,08

*p*-Werte wurden auf der Grundlage des χ^2^-Differenztests nach Satorra-Bentler berechnet (Satorra und Bentler 2001)

*χ*^*2*^ Chi-Square, *df* Freiheitsgrade, *CFI* comparative fit index, *RMSEA* root mean square error of approximation

### Known-Group-Validität

#### Makro-adaptive Lernunterstützung

Zur Analyse von Gruppenunterschieden wurden verschiedene hierarchisch-sequentielle Regressionsanalysen mit der abhängigen Variable „Qualität der makro-adaptiven Lernunterstützung“ durchgeführt. Die Aufnahme der Variable „Alter der Kinder“ führte nicht zu einer höheren Varianzaufklärung, auch dann nicht, wenn sie an erster Stelle ins Modell eingefügt wurde (*R*^*2*^ = 0,008, *p* > 0,1). Der Übersichtlichkeit halber werden die Detailergebnisse zu dieser Variable deshalb nicht dargestellt. In der ersten präsentierten Analyse wurde im ersten Schritt die Variable „Abschluss“ (akademisch, nicht akademisch) eingeführt, an zweiter Stelle der Bildungskontext bzw. das Land (Tab. 8). Die erklärte Varianz *R*^*2*^ steigt mit dem Einfügen des Prädiktors „Abschluss“ geringfügig (*R*^*2*^ = 0,07), jedoch signifikant an (*F* [1, 143] = 11,49, *p* < 0,01). Wird zusätzlich der Prädiktor „Land“ aufgenommen, führt dies zur signifikanten Veränderung von *R*^*2*^ um 0,14 (*F* [1, 143] = 25,95, *p* < 0,001).

**Table 8:** 

	Prädiktore*n*	β	*R*^*2*^	*ΔR*^*2*^	*ΔF*	*p*
Schritt 1	Abschluss	0,27	0,07	0,07	11,49	0,001
Schritt 2	Land	0,41	0,22	0,14	25,95	<0,001

Abschluss: 0 = nicht-akademisch, 1 = akademisch; Land: 0 = Deutschland, 1 = Schweiz

In Tab. 9 werden die Ergebnisse für die Analysen mit dem Prädiktor „Land“ an erster Stelle dargestellt. *R*^*2*^ steigt signifikant auf 0,21 (*F* = [1, 143] 36,88, *p* < 0,001). Das Einfügen der erklärenden Variable „Abschluss“ führt nicht zu einem signifikanten Anstieg von *R*^*2*^ (*F* [1, 143] = 2,23, *p* > 0,05).

**Table 9:** 

	Prädiktoren	β	*R*^*2*^	*ΔR*^*2*^	*ΔF*	*p*
Schritt 1	Land	0,45	0,21	0,21	36,88	<0,001
Schritt 2	Abschluss	0,12	0,22	0,01	2,23	0,138

Abschluss: 0 = nicht-akademisch, 1 = akademisch; Land: 0 = Deutschland, 1 = Schweiz

#### Mikro-adaptive Lernunterstützung

Der Known-Group-Vergleich zur Qualität der mikro-adaptiven Lernunterstützung wird in einem ersten Schritt für den fachlichen Bereich (mathematische Lernprozessanregung, mathematische Fachsprache) vorgenommen. Der Einschluss des Prädiktors „Abschluss“ führt zu einem sehr niedrigen *R*^*2*^ von 0,02 (*F* [1, 143] = 3,52, *p* > 0,05). Die Varianzaufklärung bleibt auch nach Einschluss der Variable „Land“ niedrig (*R*^*2*^ = 0,06), der Anstieg ist jedoch signifikant (*F* [1, 143] = 4,71, *p* < 0,05) (Tab. 10).

**Table 10:** 

		Fachliche Lernunterstützung					Fachunabhängige Lernunterstützung				
	Prädiktor	β	*R*^*2*^	*ΔR*^*2*^	*ΔF*	*p*	β	*R*^*2*^	*ΔR*^*2*^	*ΔF*	*p*
Schritt 1	Abschluss	0,16	0,02	0,02	3,52	0,063	0,22	0,05	0,05	7,00	0,009
Schritt 2	Land	0,19	0,06	0,03	4,71	0,032	0,52	0,28	0,24	46,76	<0,001

Abschluss: 0 = nicht-akademisch, 1 = akademisch; Land: 0 = Deutschland, 1 = Schweiz

Für die Qualität der fachunabhängigen mikro-adaptiven Lernunterstützung (Gruppenmanagement, emotionale Wärme; Tab. 10) steigt *R*^*2*^ mit dem Einschluss des Prädiktors „Abschluss“ an erster Stelle geringfügig (*R*^*2*^ = 0,05), jedoch signifikant an (*F* [1, 143] = 7,00, *p* < 0,01). Wird zusätzlich der Prädiktor „Land“ aufgenommen, führt dies zu einer signifikanten Veränderung von *R*^*2*^ von 0,24 (*F* [1, 143] = 46,76, *p* < 0,001).

Wird – wie in Tab. 11 dargestellt – die Variable „Land“ an erster Stelle eingefügt, bestätigt sich das Ergebnis des Länderunterschieds für die mikro-adaptive Lernunterstützung: Bei der fachlichen Lernunterstüzung steigt *R*^*2*^ signifikant um 0,05 (*F* [1, 143] = 7,43, *p* < 0,01), während der Einschluss der Variable „Abschluss“ zu keiner signifikanten Veränderung führt (*F* [1, 143] = 0,90, *p* > 0,05). Bei der fachunabhängigen Lernunterstützung steigt *R*^*2*^ signifikant um 0,28 (*F* [1, 143] = 56,30, *p* < 0,001), während der Einschluss der Variable „Abschluss“ zu keiner signifikanten Veränderung führt (*F* [1, 143] = 0,07, *p* > 0,05). Das heißt, dass sich hinsichtlich der fachunabhängigen Lernunterstützung entgegen den Erwartungen länderspezifische Unterschiede zeigen.

**Table 11:** 

		Fachliche Lernunterstützung					Fachunabhängige Lernunterstützung				
	Prädiktor	β	*R*^*2*^	*ΔR*^*2*^	*ΔF*	*p*	β	*R*^*2*^	*ΔR*^*2*^	*ΔF*	*p*
Schritt 1	Land	0,22	0,05	0,05	7,43	0,007	0,53	0,28	0,28	56,30	<0,001
Schritt 2	Abschluss	0,08	0,06	0,01	0,90	0,345	0,02	0,28	0,00	0,07	0,800

Abschluss: 0 = nicht-akademisch, 1 = akademisch; Land: 0 = Deutschland, 1 = Schweiz

## Diskussion

In diesem Beitrag wurde ein Messinstrument vorgestellt, mit dem in regelspielbasierten Situationen im Kindergarten die Qualität der mathematischen makro- und mikro-adaptiven Lernunterstützung (Corno und Snow 1986) gemessen werden kann, letztere mit fachabhängigen und fachunabhängigen Merkmalen. Nach Gasteiger et al. (2019) ist es wichtig, dass dabei der kindergartenspezifische bzw. frühpädagogische Kontext berücksichtigt wird. Verglichen mit bereits bestehenden Analyseinstrumenten (Abschn. 4.1) liegt die Besonderheit des Instruments erstens darin, dass die Qualität der Planung und Reflexion der mathematischen Fördersituationen als eigenständige Dimension konzeptualisiert wurde. Das Instrument zeichnet sich zweitens dadurch aus, dass bezüglich der Qualität der mikro-adaptiven Lernunterstützung sowohl die fachabhängige als auch die fachunabhängige Qualität der Lernunterstützung kindergartenspezifisch reliabel erfasst wird.

Ausgehend von der Unterscheidung der Qualität von makro- und mikro-adaptiver Lernunterstützung sowie der Trennung der mikro-adaptiven Unterstützung in eine fachbezogene und in eine fachunabhängige Dimension zeigen die Ergebnisse einer konfirmatorischen Faktorenanalyse, dass die theoretisch angenommene dreifaktorielle Struktur am besten zu den Daten passte. Das Ergebnis weist darauf hin, dass sich die Planung und Reflexion der Regelspielsituation als eine eigenständige Dimension der adaptiven mathematischen Lernunterstützung abbilden lässt. Dies ist angesichts der in den letzten Jahren verstärkt eingeführten Bildungsplänen mit konkreten mathematischen Lernzielen (z. B. DeLuca et al. 2020; Deutschschweizer Erziehungsdirektorenkonferenz 2014) bedeutsam. Davon ausgehend stellt sich für zukünftige Untersuchungen die Frage, ob die mikro- und die makro-adaptive Lernbegleitung auch separat gefördert werden können.

Allerdings stellt sich bezüglich des Vorgehens auch die Frage, inwiefern die empirische Trennbarkeit von mikro- und makro-adaptiver Lernunterstützungsqualität eventuell auf die Verwendung von unterschiedlichen Datenquellen (Videos und Interviews) zurückzuführen ist. Der Einfluss dieses Methodenfaktors konnte auf Basis der vorliegenden Daten nicht überprüft werden, da dafür zu beiden Faktoren (mikro- und makro-adaptive Lernunterstützung) Daten mit beiden Methoden hätten erhoben werden müssen. Hier zeigt sich eine grundsätzliche Herausforderung der Erhebung von Informationen zur mikro- und makro-adaptiven Lernunterstützung: Mikro-adaptive Lernunterstützung kann nur – wenn auf Selbstberichte verzichtet werden soll – durch Videoaufnahmen (oder allenfalls Beobachtung) erfasst werden. Die Planung und die Reflexion hingegen können nur durch (schriftliche oder mündliche) Berichte erhoben werden. Es kann also vermutet werden, dass zwar ein Teil des Unterschieds auf die Erhebungsmethoden zurückzuführen ist, der im Kontext dieser Fragestellung unvermeidlich ist, jedoch eine valide und informierte Interpretation der Ergebnisse dennoch möglich ist.

In der Studie wurde der Beobachtungsgegenstand der regelspielbasierten Situationen durch Vorgaben (einheitliches Spiel, vorgegebene Gruppengröße, einheitlicher Auftrag) einer gewissen Standardisierung unterworfen. Diese Vergleichbarkeit erlaubte die Analyse der Qualitätseinschätzung des Instruments über die verschiedenen Lernsituationen hinweg. Trotzdem ist die Erfassung situationsorientiert (Gasteiger et al. 2019). Allerdings müsste in weiteren Studien geprüft werden, ob das Instrument auch für die Erfassung von nicht-standardisierten Situationen eingesetzt werden kann. Ausgehend von den Erfahrungen beim Rating und dem Vergleich mit mathematikspezifischen Items aus anderen Instrumenten (z. B. Pohle et al. 2019) wird dies für spezifisch mathematische Lernsituationen, beispielsweise beim spezifisch mathematischen Freispiel „Einkaufen“, als möglich erachtet. Für sehr offene Situationen wie das Freispiel generell ist das Instrument nicht geeignet, da dort der Aspekt der gezielten Planung entfällt.

Eine weitere Zielsetzung bestand in der Überprüfung der Messinvarianz des Instruments für die beiden Gruppen der Fachkräfte in Deutschland und der Schweiz, da sich die Bildungskontexte deutlich unterscheiden (Gasteiger et al. 2020). Für die vorliegenden Daten liegt metrische Messinvarianz vor. Daraus kann geschlossen werden, dass das latente Konstrukt „Qualität der Lernunterstützung“ in den beiden Ländern die gleiche inhaltliche Bedeutung hat und die Unterscheidung der Subdimensionen in beiden Ländern vergleichbar ist. Skalare Messinvarianz, die einen itemspezifischen Vergleich erlauben würde, liegt allerdings nicht vor. Entsprechend müssen die Unterschiede zwischen beiden Ländern hinsichtlich der Qualität der Lernunterstützung vorsichtig interpretiert werden. Trotzdem sollen mögliche Gründe diskutiert werden, da dies für die Weiterentwicklung von Instrumenten zur adaptiven mathematischen Lernunterstützung bedeutsam ist.

Bei den Ergebnissen des Known-Group-Vergleichs zum Bildungskontext (Land) bildete das Messinstrument für die Schweiz die angenommene höhere Qualität der adaptiven Lernunterstützung ab. Dies steht in Übereinstimmung mit der Untersuchung von Hepberger et al. (2020), in der die mathematikspezifischen professionellen Kompetenzen von frühpädagogischen Fachkräften in der Schweiz und in Deutschland untersucht wurden. Die Unterschiede zeigten sich insbesondere bei der Qualität der makro-adaptiven Lernunterstützung, aber auch bei der fachunabhängigen mikro-adaptiven Lernunterstützung. Es wird angenommenen, dass die höhere Qualität der Lernunterstützung in der Schweiz u. a. darauf basiert, dass die Planung von Lernsituationen im stark bildungsorientierten Kindergarten in der Schweiz mit entsprechenden Planungsunterlagen und Fördermaterialien, die den Fachkräften zur Verfügung gestellt werden, stark unterstützt wird. Im Gegensatz zur Studie von Kuger und Kluczniok (2009) zeigte sich kein Einfluss des Stichprobenmerkmals Alter der Kinder. Dies könnte damit zusammenhängen, dass das Setting durch die Regelspiele stärker standardisiert war als in der besagten Studie (Erfassung der Aktivitäten durch Tagebücher).

Hinsichtlich der fachunabhängigen Lernunterstützung bestehend aus Gruppenmanagement und emotionaler Wärme können nur Vermutungen angestellt werden, weshalb die Schweizer Fachkräfte insgesamt höhere Werte aufweisen. Das Ergebnis könnte dadurch erklärt werden, dass die Schweizer Fachkräfte aufgrund der Bildungsorientierung und der strukturierten Lernsituationen stärker auf das Gruppenmanagement achten als die Kolleginnen und Kollegen in Deutschland. Hier müssten jedoch weitere Analysen folgen, die zwischen den beiden Aspekten differenzieren.

Die angenommenen Unterschiede hinsichtlich des Ausbildungsabschlusses akademisch vs. nicht akademisch bildete das Instrument nur bei der makro-adaptiven Lernunterstützung ab, wenn die Variable in einem ersten Schritt eingeführt wurde, und auch dann nur mit einem kleinen Effekt. Hier muss allerdings berücksichtigt werden, dass nur ein kleiner Teil der Fachkräfte aus Deutschland (*n* = 10 von 77) über einen akademischen Abschluss verfügten und zudem nicht immer klar war, ob es sich um einen einschlägigen fachlichen Abschluss handelt.

## Limitationen

Die Studie weist mehrere Limitationen auf. So wurden erstens die fachbezogene und die fachunabhängige Lernunterstützungsqualität im Instrument jeweils nur mit zwei Items erfasst. Der Vorteil von wenigen Items liegt in der ökonomischen Erfassung der Merkmale, da die Anforderungen an die Raterinnen und Rater steigen, je mehr Items eingeschätzt werden müssen (Fauth et al. 2014). Dennoch wäre die Untersuchung der Güte des Instruments bei Aufnahme zusätzlicher Items sinnvoll. Zweitens wurde die Qualität einer fachunabhängigen makro-adaptiven Lernunterstützung nicht untersucht. Dies ließe sich hinsichtlich des Gruppenmanagements realisieren und ist angesichts seiner Bedeutung auch im Kindergarten (Wannack und Herger 2021) ein wichtiges Desiderat für zukünftige Studien. Drittens ist die Reliabilität (Cronbach’s Alpha 0,66) für die Skala der makro-adaptiven Lernunterstützung (Tab. 6) nicht zufriedenstellend. Gasteiger et al. (2019) haben für Skalen eines Instruments zur Erfassung des „mathematical pedagogical content knowledge“ von frühpädagogischen Fachkräften in einem situationsbasierten Ansatz ähnliche Werte erhalten. Das Ergebnis der hier vorliegenden Studie könnte zum einen durch die eher offenen Fragen im Leitfadeninterview und die unterschiedlichen Kontexte in den Kindergärten (z. B. Größe der Kindergruppe) bedingt sein. Zum anderen war die Varianz der Antwortmöglichkeiten durch die Vorgabe des eher einfachen Spiels eingeschränkt. Eine Mittelung mehrfacher Messwerte zur Erhöhung der Messgenauigkeit war aufgrund der Datenlage (Interviews) für die Items zur makro-adaptiven Lernunterstützungsqualität nicht möglich. Viertens ist nicht auszuschließen, dass sich die Fachkräfte vor der Videokamera und durch die standardisierte Situation unsicher fühlten und dadurch die Lernbegleitung anders gestalteten als üblich.

Schließlich stellt sich bezüglich der Einschätzung der Qualität von Lernsituationen stets die Frage, wie viele Lerneinheiten für reliable Aussagen notwendig sind. Daten aus dem schulischen Bereich ohne Standardisierung der Lernsituation zeigen, dass für die Einschätzung des Klassenmanagements einer Lehrkraft bereits eine Lektion ausreichen kann, während insbesondere für Merkmale der kognitiven Aktivierung mehrere Lektionen erforderlich sind (Praetorius et al. 2014). In weiteren Studien müsste untersucht werden, ob sich diese Befunde auf den Kindergarten übertragen lassen.

## Ausblick

Die Analysen geben erste Hinweise dazu, dass Qualitätsmerkmale der Lernunterstützung im Kindergarten getrennt nach makro- und mikro-adaptiver Ebene sowie nach fachlichen und fachunabhängigen Aspekten gemessen werden können. Das ist ein wichtiges Ergebnis, einerseits für die Konzeptualisierung der adaptiven mathematischen Lernunterstützung, andererseits für die Entwicklung von Messinstrumenten. Für die Gestaltung von ko-konstruktiven, mathematischen Lernsituationen im Kindergarten kommt der Planung eine hohe Bedeutung zu. Einerseits scheint es deshalb sinnvoll zu sein, die Aspekte der Planungs- und Durchführungsqualität als je eigenständige Bereiche der Lernunterstützung zu fassen und dieser Unterscheidung insbesondere in der Aus- und Weiterbildung von Fachkräften Rechnung zu tragen. Andererseits zeigen die Limitationen der Studie, dass es weitere Forschungsbemühungen braucht, um diesen Aspekt der Qualität der Lernunterstützung reliabel und valide zu erfassen. Deutlich wird auch, dass der Kontext des Kindergartens im Sinne der Bildungsorientierung und der damit verbundenen Rahmenbedingungen eine zentrale Rolle zu spielen scheint. Auch das müsste in zukünftigen Untersuchungen berücksichtigt werden.

## Footnotes

1 Wir verwenden den im Schweizer Kontext gebräuchlichen Begriff „Kindergarten“ als Bezeichnung für vorschulische Bildungs- und Betreuungseinrichtungen von Kindern im Alter zwischen 4 und 6 Jahren.

2 https://www.edk.ch/dyn/12930.php.

3 Erhoben wurde, ob ein akademischer Abschluss vorlag, nicht ob ein einschlägiger akademischer Abschluss vorlag.

4 Es wurden weitere Merkmale der Fachkräfte wie das Alter und die Berufserfahrung erhoben. Da sich jedoch keine Effekte dieser Variablen zeigten, wird auf eine Darstellung verzichtet.
